# Antibiotic resistance genes in companion animals and humans driven by the gut microbial communities: composition, distribution, and implications

**DOI:** 10.1186/s12917-026-05482-z

**Published:** 2026-04-18

**Authors:** Liying Yi, Longyi An, Ruixue Wang, Xiaochen Niu, Jin Chen, Baochao Zhang, Xiaofang Pei, Xin Xu, Jiayi Chen

**Affiliations:** 1 Department of Public Health Laboratory Sciences, West China School of Public Health and West China Fourth Hospital, Sichuan University, Chengdu, Sichuan 610041 China; 2 Department of Microbiology Laboratory, Chengdu Center for Disease Control and Prevention, Chengdu, Sichuan 610041 China

**Keywords:** Antibiotic resistance genes, Mobile genetic elements, Companion animals, Microbial community, Co-occurrence network

## Abstract

**Background:**

The widespread and inappropriate use of antibiotics in both human and veterinary medicine has accelerated the emergence and dissemination of antibiotic resistance genes (ARGs) in various environments. Companion animals, due to their close and prolonged interactions with humans, have increasingly been recognized as potential reservoirs and transmitters of ARGs. However, the extent remains largely unclear to which companion animals influence the diversity and distribution of ARGs in humans. Understanding these interactions is essential for assessing environmental pathways of antibiotic resistance transmission and for developing effective mitigation strategies within the One Health framework. We examined the profiles of ARGs and gut microbial communities among three groups: companion animals, pet owners, and non-pet owners. Quantitative polymerase chain reaction (qPCR) assays were applied to determine the abundance and diversity of representative ARGs, while 16S rRNA gene sequencing was used to characterize the composition and structure of microbial communities. Comparative and correlation analyses were conducted to evaluate the relationships between ARG distribution patterns and microbial community profiles across different host groups.

**Results:**

Companion animals were found to possess the highest total abundance of ARGs (8.46 × 10^1^⁰ copies/μL), while humans exhibited greater gut microbial diversity. ARGs *ermB* and *tetQ* displayed relatively high abundance in all three groups. In addition, *intI-1* was significantly more abundant in pet owners than in non-pet owners. ARG profile of pet owners showed more similarity to that of their pets, assessed by the Jaccard similarity index. Age was associated with a limited subset of ARGs: *sul2* and *tetW* decreased with age in companion animals, whereas *aph(3’)*, *cmlA*, *fexA* and *qnrS* increased with age in humans. Notably, high correlation (r = -0.69/0.77) of *oqxA*-*Megasphaera* was identified, with negative correlation in animals and positive in pet owners, suggesting oqxA could be a potential key hub for ARGs dissemination.

**Conclusions:**

Our findings show that pet owners exhibit similar ARG profiles to those in companion animals, suggesting pet ownership may drive convergence in profiles of ARGs. Moreover, these findings provides evidence of potential resistome overlap at the human-animal interface and highlight the need to incorporate companion animals into antimicrobial control programs under a One Health framework.

**Supplementary Information:**

The online version contains supplementary material available at 10.1186/s12917-026-05482-z.

## Background

While antibiotics have drastically reduced mortality from infectious diseases, antimicrobial resistance (AMR) now presents a critical global threat to human and animal health. The core of this crisis lies in the acquisition and expression of antimicrobial resistance genes (ARGs) [1]. These genes are not only vertically inherited but also spread rapidly via horizontal gene transfer (HGT) mediated by mobile genetic elements (MGEs) like plasmids and integrons, allowing local ARGs to achieve global dissemination [2]. Therefore, it is of importance to identify the transmission of ARGs and implement surveillance and control measures for their prevalence and mutation, particularly in high-risk scenarios where the evolution and transmission of ARGs are likely to occur [3]. While the spread of ARGs has been studied in settings such as wastewater treatment plants, soil, and livestock farms [4–6], companion animals, which maintain close contact with humans, represent an emerging and critical area of concern [7].

The increase of urban companion animals may pose non-negligible AMR threat. Previous studies have reported multi-drug resistant isolates from companion animals [8]. For instance, an outbreak among companion animals in Pennsylvania caused by *Escherichia coli* carrying the *bla*_NDM-5_ carbapenemase gene, conferring resistance to nearly all β-lactam antibiotics [9]. Companion animals may also serve as reservoirs of pathogens that can be transmitted between animals and humans. Their intimate interactions with household members may facilitate the transmission of ARGs, representing a potential “One Health” risk [10]. A previous study documented the sharing of MDR *E. coli* between companion animals and their owners [11]. Various ARGs have also been identified in commensal *E. coli* strains shared between humans and companion animals, with similar distributions of integron gene cassettes [12]. Thus, in-depth research into the emergence and transmission of ARGs in companion animals is essential not only for animal health but also for safeguarding of public health.

The gut microbial community is considered a major reservoir of ARGs, and fecal excretion serves as a key pathway for their environmental dissemination [13]. Diverse and abundant ARGs have been detected in fecal samples from various companion animals [14]. Hence, the gut microbiome may be central to maintaining, disseminating, and transmitting ARGs over the long term [15]. More alarmingly, multidrug-resistant pathogens such as methicillin-resistant *Staphylococcus aureus* (MRSA) and extended-spectrum beta-lactamase-producing Enterobacterales (ESBL-E) have been reported in both humans and companion animals, raising concern about possible bidirectional exchange within households [16, 17], thereby further exacerbating the spread of resistant pathogens within gut microbial communities. Under the “One Health” framework, the gut microbial community has become a critical interface for the complex transmission of ARGs between humans and animals [18].

As a major city in southwestern China and a pet-friendly metropolis, Chengdu presents a valuable context for investigating the distribution and prevalence of ARGs. In light of this, the distribution and interactions of antibiotic resistance genes and microbial communities were investigated in companion animals, pet owners, and non-pet owners. This study provides scientific data to enhance our understanding of the transmission of ARGs and gut microbial communities between humans and companion animals.

## Methods

### Sample collection

From September to December 2024, we collected a total of 93 fecal samples: 61 from human participants (29 pet owners and 32 non‑pet owners) and 32 from companion animals. Basic characteristics of the human participants and companion animals are summarized in Table S1 and Table S2, respectively. Eligible participants and animals had no antibiotic use within the previous 6 months or gastrointestinal symptoms at sampling, companion animals had co-resided with their owners for at least 6 months; non-pet owners also reported no regular pet contact. Full inclusion/exclusion criteria and sample-level metadata are provided in the Supplementary Materials. All collected samples were properly labeled and transported to the laboratory within 4 h, where they were stored at −80 °C.

### DNA extraction and qPCR

DNA was extracted from fecal samples collected from both human participants and companion animals using the QIAamp PowerFecal Pro DNA Kit (Qiagen, Germany) in accordance with the manufacturer’s protocol. The extracted DNA was assessed for integrity, purity, and concentration using a Nanodrop spectrophotometer (Thermo Fisher Scientific, USA). Samples that passed quality control were stored at −20 °C until further analysis. Quantitative real-time polymerase chain reaction (qPCR) was performed using a real-time fluorescence-based PCR system (BIO-RAD, USA) to amplify 20 target genes [19–21]. The primer sequences, reaction mixtures, and thermal cycling conditions are detailed in Tables S4, S5, and S6. The qPCR amplification protocol and gene abundance calculations were adapted from previously published methodologies [14, 22].

### High-throughput sequencing and bioinformatics analysis

The hypervariable V3-V4 regions of 16S rRNA genes were amplified by PCR. After amplification, the PCR products were checked on a 1.5% agarose gel. Purified amplicons were then used for library construction and paired-end sequencing on the Illumina NovaSeq platform (Personalbio, Shanghai, China). All clean sequences obtained in this study were deposited in the China National Center for Bioinformation (CNCB) database with the accession number of PRJCA050714.

Raw sequencing data were processed using the QIIME2 pipeline (version 2024.5) with the DADA2 workflow for primer removal, quality filtering, denoising, merging, and chimera removal, generating amplicon sequence variants (ASVs) for downstream analyses. Taxonomic annotation of representative ASV sequences was performed against the Greengenes database (Release 13.8, http://greengenes.secondgenome.com/) [23] using the QIIME2 classify-sklearn algorithm (https://github.com/QIIME2/q2-feature-classifier). A pre-trained Naive Bayes classifier was applied in QIIME2 with default parameters to assign taxonomic labels to each feature or representative sequence. The processed amplicon sequencing data yielded microbial abundance matrices, covering multiple taxonomic levels from phylum to species.

### Network analysis

Network analysis was conducted to investigate the interactions between the gut microbial community and ARGs. The correlation matrix was constructed by calculating the Spearman's rank correlation coefficients (SRCCs) in R (version 4.5.0). A correlation between two items was identified as statistically robust if the SRCC ≥ 0.6 or ≤ − 0.6 and *p*-values ≤ 0.05. All the significant correlations were manually screened from the correlation matrix, and the network graphs were further visualized using Cytoscape 3.10.3.

### Statistics analysis

Raw experimental and sequencing data were preprocessed using Microsoft Excel. Statistical analyses were performed with SPSS software (version 22.0; IBM, USA). Group differences were assessed using the Kruskal–Wallis test combined with Monte Carlo simulation and the Fisher–Freeman–Halton test. Post-hoc comparisons were conducted using Dunn’s test with Bonferroni correction. Principal coordinate analysis (PCoA) based on Bray–Curtis distances was performed, and group differences were further evaluated using the Adonis test. Data visualization, including box plots, PCoA plots, and heatmaps, was generated using R software (version 4.5.0). Final graphical refinements were completed in Adobe Illustrator 2025 (version 29.4). A p-value of less than 0.05 was considered statistically significant in all analyses.

## Results

### Distribution of companion animals, pet owners, and non-pet owners in ARGs

A total of 93 fecal samples were analyzed, including 32 from companion animals, 29 from pet owners across 24 pet-keeping households, and 32 from non-pet owners. The experiment focused on 19 target ARGs and 1 MGE, covering tetracyclines (*tetQ, tetM, tetW, tetX*), sulfonamides (*sul1, sul2*), macrolides (*ermB, ermC*), β-lactams (*bla*_CTX-M_*, bla*_NDM_), quinolones (*oqxA, oqxB, qnrS*), phenicols (*fexA, cfr, cmlA*), aminoglycosides (*strB, aph(3’)*), polymyxins (*mcr-1*), and one mobile genetic element (*intI-1*).

**Fig. 1:**
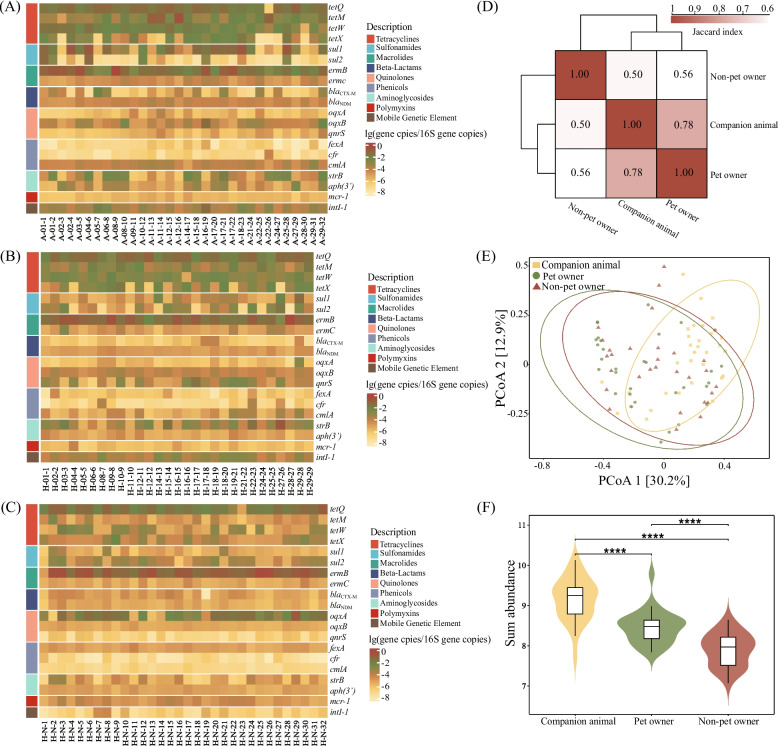
Characterization of ARGs and MGE distribution patterns and relative abundance ranges. (**A**) Distribution profile in companion animals. (**B**) Comparison of differences in total relative abundance among companion animals, pet owners, and non-pet owners. (**C**) Distribution profile in pet owners. (**D**) Heatmap of the Jaccard index for ARGs among companion animals, pet owners, and non-pet owners. (**E**) Distribution profile in non-pet owners. (**F**) Principal coordinate analysis (PCoA) of companion animals, pet owners, and non-pet owners. Note: Sample naming convention: e.g., A-01–1, H-01–1, H-N-1; where A = animal, H = human, N = non-pet owner. "01" indicates household ID, and the trailing number indicates sample ID within each group. (*P < 0.05, **P < 0.01, ***P < 0.001)

Heatmap visualization revealed clustering between companion animals and pet owners in ARG profiles (Fig. 1A, B, C). Jaccard similarity analysis further supported this pattern, showing significantly greater similarity in ARGs composition between pets and their owners (0.78) than between pets and non-pet owners (0.50) (Fig. 1D). While PCoA based on Bray–Curtis distances indicated broadly overlapping ARGs and MGE profiles between pet owners and non-pet owners, PERMANOVA confirmed significant dissimilarity among all three groups (Fig. 1E). Companion animals (8.46 × 10^1^⁰ copies/μL) generally exhibited higher ARGs and MGE abundances than pet owners (1.54 × 10^1^⁰ copies/μL) and non-pet owners (3.75 × 10⁹ copies/μL) participants (Fig. 1F).

**Fig. 2:**
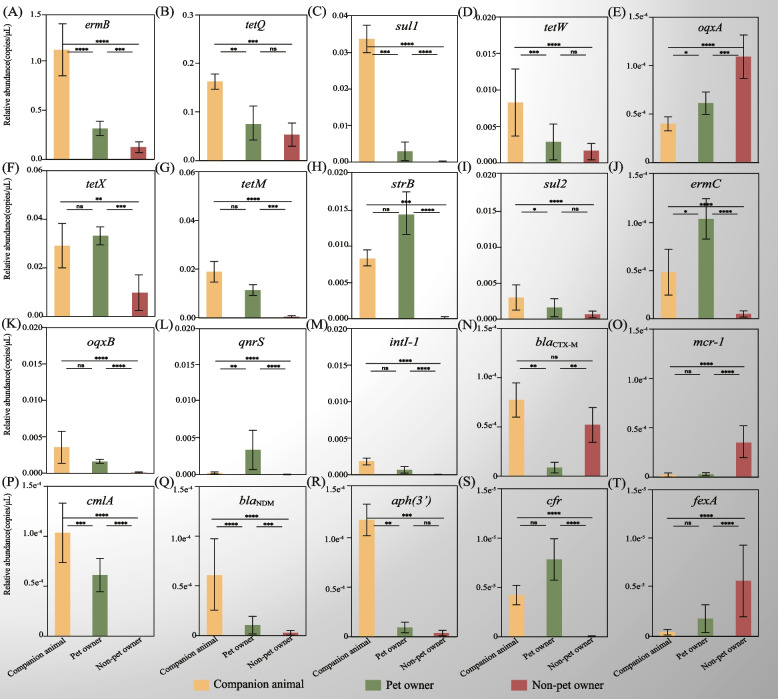
Relative abundance differences of 19 ARGs and 1 MGE in the companion animals, pet owners, and non-pet owners groups. (**A**) *ermB*, (**B**) *tetQ*, (**C**) *sul1*, (**D**) *tetW*, (**E**) *oqxA*, (**F**) *tetX*, (**G**) *tetM*, (**H**) *strB*, (**I**) *sul2*, (**J**) *ermC*, (**K**) *oqxB*, (**L**) *qnrS*, (**M**) *intI-1*, (**N**) *bla*_CTX-M_, (**O**) *mcr-1*, (**P**) *cmlA*, (**Q**) *bla*_NDM_, (**R**) *aph(3’)*, (**S**) *cfr*, (**T**) *fexA*. (The grayscale of the background represents the total abundance value of the ARGs, with darker grayscales representing lower abundance values. *P < 0.05, **P < 0.01, ***P < 0.001)

Macrolide and tetracycline resistance genes (*ermB*, *tetQ*) exhibit relatively high abundance across all groups. The macrolide resistance gene *ermB* reached the highest abundance (10⁻^2^ to 10⁻^1^ copies/μL), while most other ARGs ranged between 10⁻^3^ and 10⁻⁶ copies/μL. Non-pet owners carried most ARGs at considerably lower abundances (10⁻^5^ to 10⁻⁸ copies/μL). Differential abundance analysis identified seven ARGs (*tetM, oqxB, fexA, cfr, strB, mcr-1, intI-1*) that showed no significant difference between companion animals and pet owners, but exhibited significant differences in non-pet owners compared with both companion animals and pet owners. Three ARGs (*tetQ, sul2, aph(3’)*) did not differ significantly between pet owners and non-pet owners. The *intI-1* was particularly noteworthy, with abundance in companion animals (1.83 × 10⁻^3^ copies/μL) significantly exceeding both pet owners (6.80 × 10⁻^4^ copies/μL) and non-pet owners (1.83 × 10⁻⁶ copies/μL), post-hoc tests indicated significant differences between pets and non-pet owners, and between pet owners and non-pet owners, but not between pets and their owners (Fig. 2M).

### Effects of intrinsic factors in companion animals and humans on ARG abundance, and correlations between *intI-1* and ARGs

We performed a comparative analysis of the abundance of ARGs across different breeds, genders, and ages of companion animals. In addition, we examined the differences in ARG abundance related to the gender and age of humans.

**Fig. 3:**
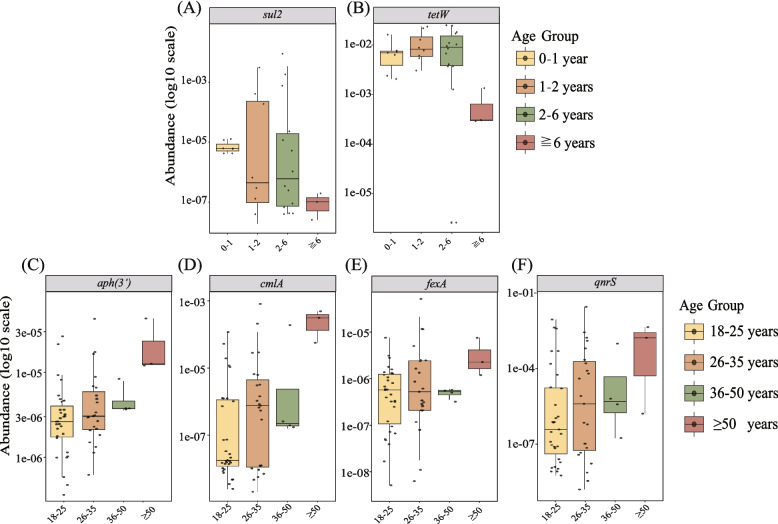
The effect of different age groups of companion animals and humans on ARG abundance. (**A**) The *sul2* gene in companion animals. (**B**) The *tetW* gene in companion animals. (**C**) The *aph(3’)* gene in humans. (**D**) The *cmlA* gene in humans. (**E**) The *fexA* gene in humans. (**F**) The *qnrS* gene in humans

**Fig. 4:**
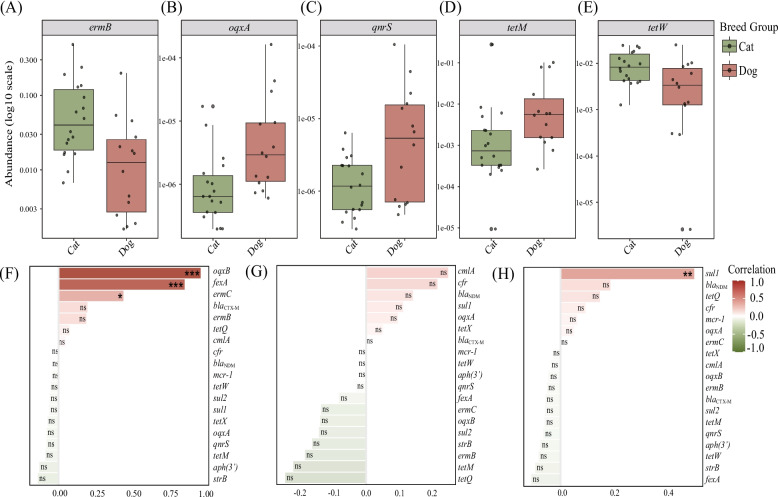
Differences in companion animal breeds significantly influence the abundance of specific ARGs. (**A**) *ermB*, (**B**) *oqxA*, (**C**) *qnrS*, (**D**) *tetM*, (**E**) *tetW*. Pearson correlation between *intI-1* and ARGs in different sample groups. (**F**) Pearson correlation analysis of companion animals. (**G**) Pearson correlation analysis of pet owners. (**H**) Pearson correlation analysis of non-pet owners. (*P < 0.05, **P < 0.01, ***P < 0.001)

Within this study, age was associated with the abundance of specific ARGs in both companion animals and humans. In companion animals, age had a significant impact on the abundance of the *sul2* and *tetW* genes (Fig. 3A, B). The abundance of the *sul2* gene exhibited a gradual decrease with increasing age. In contrast, the abundance of the *tetW* gene showed no significant variation across the 0–1 year, 1–2 year, and 2–6 year age groups but decreased markedly in animals aged ≥ 6 years. In humans, age significantly affected the abundance of four ARGs: *aph(3’)*, *cmlA*, *fexA*, and *qnrS*. A continuously increasing trend in abundance with age was observed for the *aph(3’)* and *qnrS* genes (Fig. 3C, F). For the *cmlA* gene, although a decrease in abundance was noted in the 36–50 years age group, a significant abundance peak emerged in the ≥ 50 years age group (Fig. 3D). Regarding the *fexA* gene, abundance remained relatively stable across the first three age groups but demonstrated a significant difference in the ≥ 50 years group compared to the younger humans (Fig. 3E). As shown in Table S5, no significant effect of gender on ARG abundance was observed in either companion animals or humans. (Table S5). With respect to companion animal species, significant differences in the abundance of five ARGs, *ermB*, *oqxA*, *qnrS*, *tetM*, and *tetW*, were observed between dogs and cats. Specifically, dogs generally harbored significantly higher abundances of *oqxA*, *qnrS*, and *tetM* genes compared to cats (Fig. 4B, C, D). Conversely, the abundances of ermB and tetW genes were significantly higher in cats than in dogs (Fig. 4A, E).

Furthermore, we analyzed the correlations between the *intI-1* gene and various ARGs in companion animals, pet owners, and non-pet owners, respectively. In companion animals, the *intI-1* gene showed significant positive correlations with quinolone and phenicol ARGs, with correlation coefficients all greater than 0.75. Although a significant positive correlation was also observed between *intI-1* and macrolide ARGs, the strength of this correlation was relatively weak (Fig. 4F). In pet owners, no significant correlations were found between *intI-1* and any ARGs (Fig. 4G). In contrast, among non-pet owners, a significant correlation was detected between *intI-1* and sulfonamide ARGs, though the correlation coefficient did not exceed 0.5 (Fig. 4H).

### Characteristics of gut microbial community

**Fig. 5:**
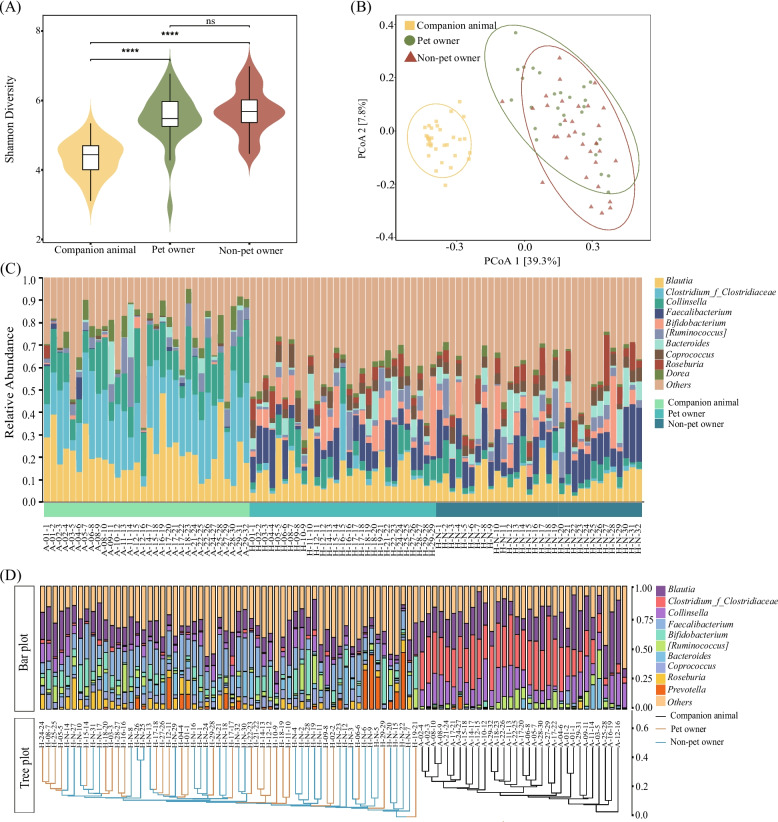
Comparison of gut microbial community relative abundance and overall diversity among companion animals, pet owners, and non-pet owners. (**A**) Shannon diversity index. (**B**) Principal Coordinate Analysis (PCoA). (**C**) Taxonomic composition. (**D**) Hierarchical clustering analysis. (*p < 0.05, **p < 0.01, ***p < 0.001)

At the genus level, clear structural differences were observed between companion animals and human samples, with the most dominant genera belonging to the phylum *Firmicutes* (Figure S1A). In companion animals, *Clostridium-f-Clostridiaceae* was the predominant genus, with relative abundances ranging from 1.4% to 51.5%. *Collinsella* (1.6%–33.4%) and *Blautia* (6.8%–48.4%) were also identified as core genera in this group. In contrast, *Faecalibacterium* (1.5%–29.5%), *Bifidobacterium* (0.2%–35.5%), and *Roseburia* (0.5%–22.5%) were the major genera in both pet-owning and non-pet-owning human groups (Fig. 5C). LefSe analysis further highlighted differentially abundant taxa across groups, revealing several indicator genera (LDA > 2.0, *P <* 0.05) for each group (Figure S1B).

We observed that companion animals exhibited significantly lower Shannon diversity compared to human samples, while no notable difference was detected between pet owners and non-pet owners (Fig. 5A). Principal coordinate analysis revealed statistically significant differences in microbial community structure among all three groups (Fig. 5B). Jaccard similarity analysis indicated that the gut microbiome similarity between companion animals and pet owners (0.46) was significantly higher than that between companion animals and non-pet owners (0.43). However, the highest level of similarity was observed between the two human groups (0.71) (Figure S1C). Analysis of Amplicon Sequence Variants (ASVs) showed that pet owners harbored 4,218 unique ASVs, while non-pet owners exhibited a greater number of unique ASVs (7,037). Notably, the number of microbial taxa shared between pet owners and companion animals (153) was substantially higher than that between non-pet owners and companion animals (53). Nevertheless, the number of shared taxa between the two human groups remained the highest (848) (Figure S1D). Interestingly, companion animal samples from the same household showed high consistency in genus-level composition, as illustrated by sample pairs A-01–1 and A-01–2, A-02–3 and A-02–4, and A-08–9 and A-08–10 (Fig. 5D).

### The microbial community mainly affects the variation of ARGs

**Fig. 6:**
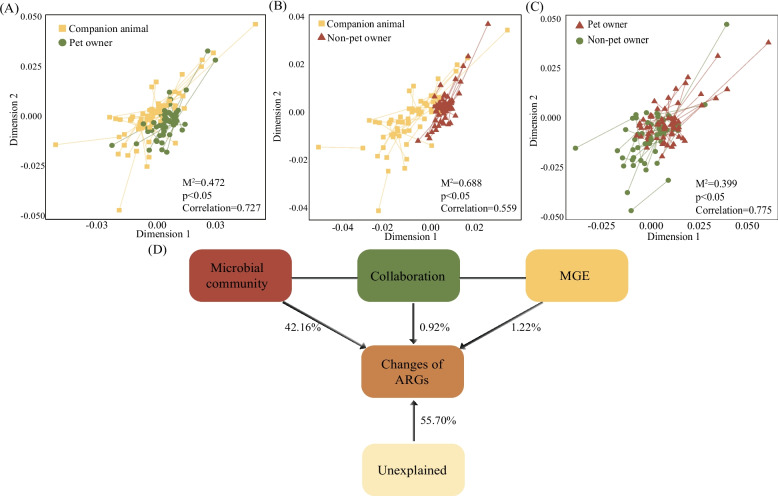
Correlation analysis of ARGs/MGEs and microbial community based on Procrustes analysis. (**A**) Procrustes analysis of companion animals and pet owners. (**B**) Procrustes analysis of companion animals and non-pet owners. (**C**) Procrustes analysis of pet owners and non-pet owners. (**D**) Variation partitioning analysis (VPA) differentiates the effects of microbial community and MGEs on the changes of ARGs

Procrustes tests were employed to determine whether the differences in ARGs among the three groups, companion animals versus pet owners, companion animals versus non-pet owners, and pet owners versus non-pet owners, were driven by microbial community composition (Fig. 6A, B, C). Significant correlations between ARG abundance and microbial community structure were detected in all three comparisons. However, the correlation strength between companion animals and non-pet owners was notably weaker than that observed in the other two groups(companion animals, pet owners, and pet owners, non-pet owners). Although statistically significant (*P <* 0.05), the microbial community explained a smaller proportion of the variance in ARG profiles in this group, suggesting the potential involvement of additional influencing factors. These results indicate that while microbial communities drive ARG distribution across all three groups, their explanatory power is substantially lower in the companion animal–non-pet owner comparison.

Furthermore, variation partitioning analysis (VPA) was performed to quantify the contributions of microbial communities and mobile genetic elements (MGEs) to the observed variation in ARGs (Fig. 6D). The model explained 44.30% of the total variance in ARG profiles. Microbial communities accounted for 42.16% of the explained variance, while MGEs contributed 1.22%. The shared effect between both factors was minimal (0.92%), indicating that microbial community composition serves as the dominant driver of ARG distribution differences between companion animals and humans in this study.

###  Potential host analysis of ARGs and MGEs

**Fig. 7:**
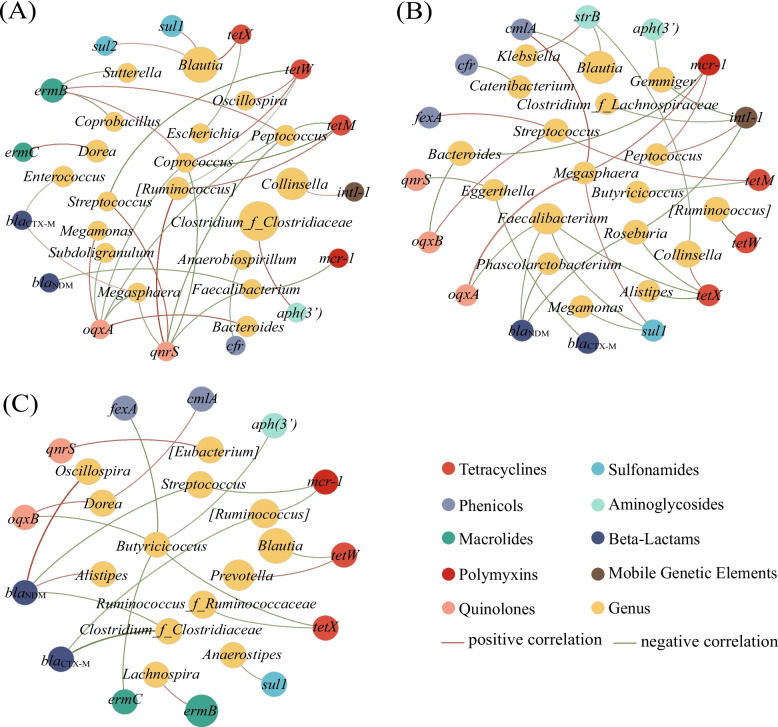
Co-occurrence network analysis of ARGs/MGE and gut microbial community. (**A**) Analysis of the co-occurrence network of companion animals. (**B**) Network analysis of co-occurrence among pet owners. (**C**) Network analysis of co-occurrence among non-pet owners. (Node size represents the relative abundance of ARGs or bacterial taxa, while edge color intensity indicates correlation strength. All correlations shown are statistically significant at *P <* 0.05.)

Given the distinct microbial community structures observed in fecal samples from companion animals and human populations, we hypothesized that the potential hosts of ARGs would differ among the three groups. Accordingly, this study analyzed correlations between 19 ARGs, one MGE, and the top 30 most abundant bacterial genera. A robust co-occurrence relationship was defined as a Spearman's correlation coefficient (|Rs|) > 0.6 with a statistical significance of *P <* 0.05. Microbial taxa satisfying these criteria were considered potential hosts of the corresponding ARGs. This stringent threshold helped exclude weakly supported or spurious associations, thereby enhancing the biological relevance of the inferred network and reducing interference signals arising from low-abundance taxa or ARGs. Network analysis revealed higher complexity in both the companion animal group (34 nodes, 35 edges) and pet owners (34 nodes, 34 edges) compared to non-pet owners (26 nodes, 21 edges) (Fig. 7).

In companion animals, *qnrS* and *oqxA* exhibited significant correlations with six and five bacterial genera, respectively, indicating their major roles in the transmission of antibiotic resistance. *Coprococcus* was linked to five ARGs, suggesting its potential as a key reservoir of ARGs in companion animals. However, no significant correlation was observed between *Coprococcus* and the *intI-1* gene. Additionally, *intI-1* displayed a specific positive correlation with *Collinsella*, a dominant genus in companion animals (Fig. 7A). In the pet owner network, genera including *Megasphaera*, *Streptococcus*, and *Peptococcus* were positively correlated with several ARGs. *IntI-1* was also associated with four microbial genera, among which *Peptococcus* showed a positive correlation (Fig. 7B). In contrast, the network for non-pet owners was sparser, with most ARGs being either isolated or negatively correlated with bacterial taxa. Notably, *Butyricicoccus* was negatively correlated with multiple ARGs, such as *tetX* and *oqxB*, whereas *Dorea* was positively correlated with *oqxB* and *cmlA*. The carbapenem resistance gene *bla*_NDM_, which was not prominent in the other two groups, emerged as a key node among non-pet owners and was strongly correlated with *Oscillospira*. *IntI-1* showed no correlations in this group, a finding distinct from the patterns observed in companion animals and pet owners (Fig. 7C).

Cross-group comparisons highlighted that *Blautia*, a core gut genus, was consistently correlated with ARGs across all groups, though the direction of correlation was population-specific. In human samples, it was largely negatively correlated with ARGs, whereas in companion animals it showed positive correlations with *sul1*, *sul2*, and *tetX *(Fig. 7A, B, C). Similarly, *Faecalibacterium* was negatively associated with most ARGs in networks from companion animals and pet owners (Fig. 7A, B), and *Butyricicoccus* consistently showed negative correlations with ARGs in both human groups (Fig. 7B, C). *Streptococcus* was present in all three networks but correlated positively with quinolone resistance genes in pets and pet owners, and negatively with *bla*_NDM_ in non-pet owners (Fig. 7A, B, C). Of particular interest was the identification of an *oqxA–Megasphaera* association in both the companion animal and pet owner networks. However, the correlation was negative in companion animals and positive in pet owners. No such association was detected in non-pet owners (Fig. 7A, B).

## Discussion

In this study, the profiles of ARGs and MGEs and gut microbial communities across three groups were studied. Our findings highlight the role of companion animals as significant reservoirs of ARGs and indicate their potential influence on the antimicrobial resistome of humans in close contact.

The fact that all targeted ARGs were detected in all 93 samples suggests that antimicrobial resistance is highly prevalent and widely disseminated [24]. The high abundance of macrolide and tetracycline ARGs is consistent with previous observations, indicating that tetracycline and macrolide ARGs consistently dominate the resistome in both human and animal gut microbial communities [25–27]. The pervasive presence of these resistance determinants may be linked to the frequent use of antibiotics in human medicine and the common off-label use of human-grade antibiotics in veterinary practice [28]. Furthermore, the high abundance of tetracycline- and macrolide-resistance genes may reflect the widespread use of these antibiotic classes in human and veterinary settings and the potential for bioaccumulation [29]. Clinical observations indicate that companion animals often undergo prolonged antibiotic regimens for chronic conditions such as dermatological and respiratory infections, which may contribute to the selective enrichment of these specific ARGs. Notably, the class 1 integron gene (*intI-1*), a key genetic marker associated with HGT and an indicator of anthropogenic antibiotic pollution [30], particularly exhibits the highest levels in companion animals, suggesting that cohabitation with pets contributes to the convergence of ARGs profiles between humans and companion animals. Meanwhile, the *intI-1* may facilitate cross-species transmission through frequent human–animal contact [31].

More importantly, our study showed that companion animals harbored a significantly higher abundance of ARGs than humans, suggesting that they may represent an important reservoir context for ARG exposure, which is consistent with previous reports [10]. In addition, ARG profiles in companion animals were more similar to those of pet owners than to those of non-pet owners. Previous studies have shown that occupational exposure to livestock-associated environments can reshape human ARG profiles [32, 33], and other studies have reported greater similarity in ARG and MGE profiles between domestic dogs and their owners [26, 34]. Taken together, our findings suggest that close contact associated with pet ownership may increase opportunities for ARG overlap between companion animals and humans and may therefore be associated with variation in the human gut resistome.

Among the β-lactam resistance genes detected in this study, *bla*_CTX-M_ merits particular attention. Retrospective evidence suggests that *bla*_CTX-M_ may have predated its first recognized clinical report, which was first isolated in 1986 from a laboratory dog [35]. Since then, *bla*_CTX-M_ have been reported in Germany, South America and all over the world in humans, animals, and the environment [35–39]. Over the following decades, numerous variants of *bla*_CTX-M_ have been identified, particularly in companion animals and poultry, highlighting its continued dissemination and genetic diversification [40, 41]. In this study, *bla*_CTX-M_ was detected in all three study groups with relatively higher abundance in companion animals, suggesting that its occurrence may reflect broader community circulation. Therefore, long-term monitoring of *bla*_CTX-M_ remains indispensable, and genome-based surveillance across human–animal–environment interfaces is needed to clarify its transmission pathways.

The dissemination of ARGs in the gut is influenced by multiple host and environmental factors, including antibiotic exposure, disease status, age, and diet [42]. A better understanding of how these factors relate to ARG profiles may help enact strategies to mitigate AMR. With regard to age, some studies have suggested that human aging appears to be associated with an increased burden of ARGs, consistent with the trend of increased abundance that we observed from several ARGs, including *aph(3’)*, *cmlA*, *fexA*, *qnrS* [43]. However, age-related patterns in ARG abundance may vary across host species and study settings. In livestock, for example, younger animals have sometimes been reported to harbor higher ARG abundance than older ones [44, 45]. In our study, only two ARGs, *tetW* and *sul2*, showed significantly higher abundance in young companion animals than in older ones. These findings suggest a possible age-associated difference for selected ARGs in companion animals. Further studies are still needed to clarify the potential impact of host age on the gut resistome.

Moreover, the microbial community contributed substantially to the total variation in ARGs, while MGEs and their joint effects played relatively minor roles. Multiple researchers have also indicated that bacterial abundance is the primary positive factor driving ARG patterns [46, 47]. In contrast, Yu et al. reported that in wastewater treatment systems, the microbial community (24.7%) contributed less to ARGs variation than MGEs (32.2%) [48]. This discrepancy may be attributed to differences in the studied environments, highlighting the contextual variability of such relationships. Therefore, our study on companion animals and human populations suggests that the microbial community remains a key and dominant driver of ARG variation.

Distinct correlation patterns between ARGs, MGE, and their associated bacterial hosts were observed across human and companion animals, indicating that host ecological niche and lifestyle may play determining roles in shaping the distribution and transmission pathways of ARGs [49]. Comparisons across three co-occurrence networks suggest that pet ownership may enhance the strength of interactions between gut microbial communities and ARGs. Furthermore, the co-occurrence of *Coprococcus* and *Peptococcus* with *ermB* in companion animals suggested that these genera may participate in macrolide-resistance-associated microbial consortia. Previous studies have detected *ermB* in *Enterococcus spp.* from companion animals [50], supporting the relevance of macrolide-resistance determinants in pet-associated microbiota. Likewise, the association of *Collinsella* with ARGs in companion animals and pet owners suggested that this genus deserves further investigation in host-ARG interaction networks.

The* oqxA* and *qnrS* were widely detected across all groups and were correlated with multiple genera in companion animals, suggesting that these quinolone resistance genes were broadly distributed within the observed gut microbial association network. In this study, *Megasphaera* showed positive correlations with multiple ARGs and *oqxA* was positively associated only with *Megasphaera* in pet owners. *Megasphaera* is a strictly anaerobic, lactate-utilizing common inhabitant that has been isolated from pigs, ruminants, and humans [51–54]. Genome analyses showed *Megasphaera* encodes for genes that confer resistance to quinolones, β-lactams and other specific antibiotics [55, 56], suggesting that it may serve as an important host for quinolone resistance genes. Besides, mobile genetic elements such as transposons were also detected in *Megasphaera* [57], indicating its potential in the dissemination of ARGs. Interestingly, *Megasphaera* was negatively correlated with *oqxA* in companion animals. *Megasphaera* isolates displayed differential adaptive features for survival in the gut [58]. Thus, the difference in this association may be attributed to differences in diet and environment between humans and companion animals, consequently suggesting differences in host-specific gut ecological niches, and competition with potential bacterial populations under different metabolic conditions [59, 60]. Follow-up studies integrating culture-based isolation and genome-resolved localization analyses in companion animals will be needed.

This study also has limitations due to a cross-sectional survey and a relatively small sample size. Expanding sample types, such as saliva, skin, and including more families, may better describe the distributions of ARGs in companion animals and humans. Besides, applying a longitudinal design may better track the dynamic changes of ARGs and help clarify the potential influencing factors.

## Conclusion

This study investigated the role of the gut microbial communities in shaping ARG profiles by analyzing their distribution across companion animals, pet owners, and non-pet owners. Our findings demonstrate that pet ownership drives a convergence of ARG profiles between owners and their companion animals. The host-specific association between *oqxA* and *Megasphaera* highlights this gene–taxon pair as a potential focus for future studies on ARG transmission. This study underscores the impact of cohabitation with pets on the gut resistome and microbiome. It is imperative to integrate companion animals into AMR surveillance and to enforce prudent antibiotic use in veterinary practice, so as to safeguard global health security and mitigate the threat of antimicrobial resistance.

## Supplementary Information

Supplementary Material 1: Table S1. Baseline characteristics of humans. Table S2. Baseline characteristics of companion animals. Table S3. Detailed information about all metagenome samples. Table S4. Primer information for quantification of antibiotic resistance genes (ARGs) in companion animal and human fecal samples. Table S5. qPCR reaction mixture components. Table S6. qPCR cycling conditions. Table S7. Differences in relative abundance among companion animals with different biometric characteristics, pet owners, and non-pet owners.

Supplementary Material 2: Figure S1. The additional microbial community characteristics of companion animals, pet owners, and non-pet owners.

## Data Availability

All data from this study are available from the corresponding author upon request. The datasets presented in this study can be found in online repositories. The names of the repository/repositories and accession numbers can be found below: https://ngdc.cncb.ac.cn/bioproject/browse/PRJCA050714.

## Abbreviations

AMR: Antimicrobial resistance
ARB: Antibiotic-resistant bacteria
ARGs: Antibiotic resistance genes
HGT: Horizontal gene transfer
MGE: Mobile genetic elements
