# Supplementary material for: Antibiotic resistance genes in companion animals and humans driven by the gut microbial communities: composition, distribution, and implications
Source: BMC Vet Res. 2026 Apr 18;22:357. doi: 10.1186/s12917-026-05482-z (PMC13281641; doi:10.1186/s12917-026-05482-z)
Supplement: Supplementary file 2 — Supplementary Material 2: Figure S1. The additional microbial community characteristics of companion animals, pet owners, and non-pet owners. [file 12917_2026_5482_MOESM2_ESM.docx]

Supplementary Material for

**Antibiotic Resistance Genes in Companion Animals and Humans Driven by the Gut Microbial Communities: Composition, Distribution, and Implications**

**Liying Yi ^1 a^, Longyi An ^2 a^, Ruixue Wang** **^1^, Xiaochen Niu ^1^, Jin Chen ^1^, Baochao Zhang ^1^, Xiaofang Pei ^1^, Xin Xu ^1^, Jiayi Chen ^1^** *

^1^ Department of Public Health Laboratory Sciences, West China School of Public Health and West China Fourth Hospital, Sichuan University, Chengdu 610041, Sichuan, China;

^2^ Department of Microbiology Laboratory, Chengdu Center for Disease Control and Prevention, Chengdu 610041, Sichuan, China;

*Corresponding Author

Postal address: West China School of Public Health and West China Fourth Hospital, Sichuan University, Chengdu, 610041, China.

Tel: 86-15208206151 (Jiayi Chen)

E-mail address: cjy.210@163.com (Jiayi Chen)

a These authors contributed equally to this work.

**Inclusion and Exclusion Criteria for Pet Owners:**

(1) Age ≥ 18 years and written informed consent provided.

(2) No antibiotic use within the previous 6 months.

(3) No symptoms of intestinal disorders (for example, diarrhea or constipation) within the previous 3 days.

**Inclusion and Exclusion Criteria for Companion Animals:**

(1) The companion animal had lived with the owner for at least 6 months.

(2) Neither the companion animal nor the cohabiting owner had used antibiotics within the previous 3 months.

(3) Neither the companion animal nor the owner had experienced recent intestinal symptoms (for example, diarrhea or constipation) within the previous 3 days.

**Inclusion and Exclusion Criteria for Non-Pet Owners:**

(1) No pet ownership history in the household during the previous 2 years (primarily cats or dogs).

(2) No regular direct pet contact during the previous 6 months, including petting, feeding, or cleaning pet waste.

(3) No frequent exposure to pet-associated environments during the previous 6 months (for example, veterinary clinics, pet stores, boarding facilities, or waste processing sites).

(4) No antibiotic use within the previous 6 months.

(5) Age ≥ 18 years and written informed consent provided.

(6) No diarrhea or constipation within the previous month.

Table S1. Baseline characteristics of humans

| Characteristic | Pet owners | Non-pet owners |
| --- | --- | --- |
| Number, n | 29 | 32 |
| Male, n (%);  Female, n (%) | 6 (20.7%);  23 (79.3%) | 6 (18.8%);  26 (81.2%) |
| Age (years),  median [IQR] (range) | 27 [24–29] (22–60) | 25 [25–30] (20–50) |
| Co-residence with current companion animal (years),  median [IQR] (range) | 1.75 [0.96–3.00] (0.5–13.0) | - |

Table S2. Baseline characteristics of companion animals

| Characteristic | Companion animals |
| --- | --- |
| Number, n | 32 |
| Species, n (%) | Cats 18 (56.3%);  Dogs 14 (43.8%) |
| Male, n (%);  Female, n (%) | 17 (53.1%);  15 (46.9%) |
| Age (years),  median [IQR] (range) | 2.0 [1.1–4.0] (0.6–13.0) |
| Co-residence with current pet-owners (years),  median [IQR] (range) | 1.75 [0.96–3.00] (0.5–13.0) |

Figure S1: Additional gut microbial community characteristics of companion animals, pet owners, and non-pet owners. (A) Sankey diagram of the gut microbiome at the phylum, class, order, family, and genus levels. (B) LEfSe analysis of biomarker taxa among the three groups. (C) Heatmap of the species-level distance matrix. (D) Shared species among the three groups.
